# Acute Stress Shapes Creative Cognition in Trait Anxiety

**DOI:** 10.3389/fpsyg.2019.01517

**Published:** 2019-08-08

**Authors:** Haijun Duan, Xuewei Wang, Zijuan Wang, Wenlong Xue, Yuecui Kan, Weiping Hu, Fengqing Zhang

**Affiliations:** ^1^MOE Key Laboratory of Modern Teaching Technology, Shaanxi Normal University, Xi’an, China; ^2^Jinyuan International School, Shaanxi Normal University, Xi’an, China; ^3^Collaborative Innovation Center of Assessment Towards Basic Education Quality, Beijing Normal University, Beijing, China; ^4^Department of Psychology, Drexel University, Philadelphia, PA, United States

**Keywords:** acute stress, creative cognition, Trier social stress test, alternative uses test, remote associates test

## Abstract

This study examined the cognitive mechanism underlying acute stress in creative cognition among individuals with high and low trait anxiety. Specifically, cognitive inhibition was assessed using the flanker task during acute stress. Fifty-two participants (26 with high trait anxiety, 26 with low trait anxiety, with a mean age of 18.94 years) underwent stress induction *via* the Trier Social Stress Test (TSST). They all completed the Alternative Uses Test (AUT) and the Remote Associates Test (RAT) before and after the TSST. Biochemical markers (salivary cortisol and salivary alpha amylase) were recorded at regular intervals. The results showed that cognitive inhibition was influenced by trait anxiety and acute stress. In low-trait anxious individuals after experiencing acute stress, there was a lack of cognitive inhibition and they performed better in AUT (fluency), compared to before experiencing acute stress, whereas high-trait anxious individuals showed a decreased interference effect and reduced performance in AUT (fluency, flexibility, and originality). In the RAT, there were shorter response times and increased accuracy after acute stress in both high- and low-trait anxiety groups. Thus, we suggest that cognitive control, which modulates changes in acute stress, influences creative cognition. These findings provide evidence that inhibition control mediates the effect of stress on the creativity of individuals with different trait anxiety.

## Introduction

Creativity has long been of great interest in a wide range of fields. People have the ability to exert cognitive control over creativity (Beaty et al., 2014; Kenett et al., 2018). According to controlled-attention theory, creative production depends on individuals’ ability to exert control over their attention and cognition (Gilhooly et al., 2007; Beaty et al., 2016). Individuals high in creative thinking tend to be able to dynamically change their level of control according to the current task requirements (Gilhooly et al., 2007; Radel et al., 2010; Benedek et al., 2012). However, our ability to control creativity is not always consistent, especially in the face of suddenly occurring situations.

Stress is an unavoidable feature of modern life. Stress activates the sympathetic nervous system (SNS) and hypothalamus-pituitary adrenal (HPA) axis. In human saliva, the activity of the SNS and HPA can be measured by salivary alpha amylase (sAA) and salivary cortisol (sC), respectively (Kirschbaum and Hellhammer, 1989; Foley and Kirschbaum, 2010). It is vital that individuals have the ability to exert cognitive control in the face of stress—not doing so may cause stress to impair functioning (Erskine et al., 2007), causing serious distress and mental impairment (McNally, 2006; Qureshi et al., 2011; Cisler and Olatunji, 2012).

Previous literature has presented inconsistent results regarding the association between stress and creativity. Some have found that stress leads to a decrease in creativity (Beversdorf et al., 1999; Probst et al., 2007; Byron et al., 2010; Lovelace and Hunter, 2013; Duan et al., 2019; Wang et al., 2019), while others found that it increased creativity (Baas et al., 2008; Ohly and Fritz, 2010). Still others found a U-shaped relationship between stress and creativity (Suedfeld and Vernon, 1965; Baer and Oldham, 2006; Yeh et al., 2015). Meanwhile, trait anxiety was usually considered as a stress-vulnerability factor (Eysenck and Derakshan, 2011; Berggren and Derakshan, 2013; Ward et al., 2017; Weger and Sandi, 2018).

Trait anxiety has been defined as the tendency of individuals to experience frequent and high-intensity anxiety and worry in the face of stressful situations (Spielberger, 1979). Hence, studies could focus on highly anxious individuals, who are more prone to anxiety in stressful situations. Additionally, these past studies did not explore the cognitive mechanisms underlying how stress affects creativity. As mentioned above, completing creative activities and facing stress both require cognitive control. Thus, the core mechanism underlying creative generation under stress may be an executive control process, including the ability to inhibit the influence of irrelevant information caused by stress. However, this fascinating possibility remains to be addressed.

According to the Attentional Control Theory (ACT; Eysenck et al., 2007), anxiety activates the stimulus-driven system and reduces the goal-driven system (the crux of the ACT is that we have two attentional systems: top-down, goal-driven processing and bottom-up, stimulus-driven processing). Individuals with high trait anxiety (HTA) appear to be more affected by stimulus-driven processing and find it difficult to suppress threatening stimuli from entering attention. Indeed, in the face of threat stimuli, individuals with HTA show more pronounced cognitive deficits, including weakened dorsolateral prefrontal cortex (DLPFC) activity (Clarke et al., 2014; Greening and Mitchell, 2015). Hence, individuals with HTA appear to show both behavioral alterations and cognitive deficits. Further research has revealed that anxiety affects the processing efficiency of executive functioning (shifting, updating and inhibition) when faced with threatening information (e.g., stress; Navarro et al., 2012; Edwards et al., 2015; Goette et al., 2015; Fogelman et al., 2016; Fonzo and Etkin, 2017). Thus, anxiety is related to a diminished ability to inhibit threat (Cisler and Koster, 2010).

A group of researchers suggested that acute stress may affect core executive functions (Hermans et al., 2014; Shields et al., 2016; Gu et al., 2018), particularly inhibition control (Shields et al., 2016). However, there are scant empirical studies examining the effect of acute stress on inhibition control in individuals with HTA and low trait anxiety (LTA). Some indirect evidence from studies on cortisol and individual differences in anxiety implied that anxiety-related personality traits modulate cognitive control processes under stress (Edwards et al., 2017). On the one hand, Grillon et al. (2017) asked participants to perform inhibition tasks while under threat of an electric shock, and found that increased anxiety promoted inhibition control. Individuals needed to allocate only a small amount of attentional resources to fully process the task-related information (Chajut and Algom, 2003). They explained the results using attention approach theory, positing that HTA individuals may have more limited attentional resources than LTA individuals, and thus their available attentional resources may become exhausted quickly under stressful conditions. On the other hand, according to the ACT (Eysenck et al., 2007), HTA individuals could display worse inhibition control under stress (Cisler and Koster, 2010; Navarro et al., 2012; Edwards et al., 2015; Goette et al., 2015; Fogelman et al., 2016; Fonzo and Etkin, 2017).

The relationship between cognitive inhibition and creativity has been explored in relation to creativity. Researchers hold that there are two types of creative thinking: convergent and divergent thinking. Convergent thinking involves deriving a single correct solution, while divergent thinking involves thinking of as many potential solutions as possible. For convergent thinking, high inhibitory control is necessary to prevent irrelevant ideas from entering into working memory and helping individuals focus on identifying solutions that meet the required standards (Zhou et al., 2019). However, for divergent thinking, low inhibition (involving automatic association and a lack of filtering of seemingly irrelevant information) may actually facilitate generation of creative ideas (Chrysikou et al., 2014; Barr et al., 2015; Radel et al., 2015; Beaty et al., 2016; Hao et al., 2016; Kenett et al., 2018).

The present study drew on previous research topics involving the complex interaction between cognitive control, stress, and creativity, with a particular focus on individual differences in trait anxiety. To date, no study has considered all four of these variables together. The present study examined the relationships among trait anxiety, acute stress, and inhibitory control using a version of the flanker task. Trait anxiety was operationalized using the Chinese version of the trait anxiety portion of the State–Trait Anxiety Inventory (STAI; Spielberger et al., 1983; Shek, 1993). Acute stress was induced using the Trier Social Stress Test (TSST) (Kirschbaum et al., 1993; Kudielka et al., 2007). This procedure allowed us to investigate whether trait anxiety under stress affects individuals’ ability to exert inhibitory control to influence creative performance outcomes. To summarize, we explore the difference in creativity between HTA and LTA individuals who underwent a stressful situation, and determined if inhibitory control mediated the effect of acute stress on creativity. To this end, two hypotheses were formulated: (1) if acute stress impairs inhibitory control processes, HTA individuals will present better divergent thinking performance and worse convergent thinking performance; (2) if acute stress does not impair inhibitory control processes, HTA individuals will present worse divergent thinking performance and better convergent thinking performance.

## Materials and Methods

### Subjects

Initially, 713 undergraduate students from Shaanxi Normal University (pre-test) completed the Chinese version of the STAI. Based on their scores, we chose individuals for the HTA group (upper 27th percentile of the distribution) and LTA group (lower 27th percentile). In the present study, we invited 52 individuals, including 26 HTA individuals (*M*_age_ = 18.46 years, SD = 0.89) and 26 LTA individuals (*M_age_* = 19.42 years, SD = 1.31), to participate. An independent-samples *t*-test revealed that the HTA group had higher trait anxiety scores (*M* = 55.42, SD = 5.85) than did the LTA group (*M* = 35.58, SD = 8.04) at pre-test, *t*(50) = −9.976, *p* < 0.001. We applied the following criteria when selecting these participants. We excluded those with a body mass index (BMI) below 18 or exceeding 27 kg/m^2^; those engaged in drug use; those who regularly consumed coffee or alcohol; and those with chronic or acute illnesses. Furthermore, participants were advised to refrain from physical exercise and consumption of food and drinks, except water, 3 h before the test sessions began (Kuhlmann et al., 2005; Kudielka et al., 2009). Female participants were not menstruating. The experiment was conducted from 2:00 pm to 5:00 pm owing to the circadian rhythms (Izawa et al., 2010). The study conformed to the principles of the Declaration of Helsinki (World Medical Association, 2013) and was approved by the Academic Committee of the Ministry of Education of Key Laboratory of Modern Teaching Technology, Shaanxi Normal University in China. All participants provided written informed consent after the procedures were fully explained, and were paid for their participation in the study.

### Experiment Procedure

**Figure 1:**
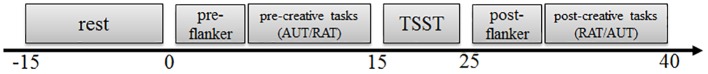
Schematic illustration of the procedure. Saliva from the participants was collected at five time points (−15, 0, 15, 25, and 40 min in relation to the onset of the stressful task). After a rest phase, participants performed Flanker task and creative tasks (AUT and RAT) before and after the TSST.

To control for individual differences, this study used a mixed design, with time of measurement (pre-test, post-test) as a within-subjects factor (McHugh et al., 2010) and group (HTA, LTA) as the between-subjects factor. The dependent variable of this experiment was performance on two creative thinking tasks: the Remote Association Test (RAT) and the Alternative Uses Test (AUT). The indicators selected for the RAT were response time and accuracy, and those for the AUT were fluency, flexibility, and originality. The overall procedure was as follows: the first salivary sample (S1) was collected on participants’ arrival at the laboratory. Subsequently, they completed a questionnaire on their demographic information, followed by the STAI. The subjects were allowed to relax for 15 min before the second salivary sample (S2). Participants in the HTA and LTA groups then completed the pre-test tasks (flanker and creative tasks) for 15 min, and the TSST for 10 min. After the TSST, the third salivary sample (S3) was taken. Then, all participants performed the post-test tasks (flanker and creative tasks). The order of the creative tasks was counterbalanced across participants. The AUT and RAT were administered *via* a computer using the E-Prime 2.0 software (Psychology Software Tools, Inc., Sharpsburg, Pennsylvania, USA) (see Figure 1).

#### Stress Task

According to the procedure of the TSST (Kirschbaum et al., 1993; Kudielka et al., 2007), participants were asked to create a 5-min interview speech for applying to college, which they would deliver to a panel of college counselors. They were given 3 min for preparation. The panel consisted of two experimenters. If a participant’s speech did not reach the full 5 min, they had to answer questions given by the experimenters until the full 5 min had passed. Finally, participants were asked to orally report answers to arithmetic problems (they had to subtract increments of 17 from 2023) as quickly and as accurately as possible for 5 min. When they made an error, the experimenter interrupted and instructed the participant to start over at 2023. The entire stress task was recorded with a digital video camera. Experimenters maintained a cold and reserved manner throughout.

#### Flanker Task

The flanker task measures individuals’ ability to selectively attend to a target and ignore distractors (Eriksen and Eriksen, 1974). According to Friedman and Miyake (2004), inhibitory control has at least two components: inhibition of the dominant response and prevention of distracting interference. Compared to other inhibition tasks, the flanker task is considered to best reflect an individual’s ability to engage in inhibition control (Redick and Engle, 2006; Shields et al., 2016). In the flanker task, a central arrow (1.48° × 0.82°) was flanked by two distractor arrows, which were kept at a distance of 0.16°. The distractor arrows were pointed either in the same (i.e., congruent trial) or opposite directions (i.e., incongruent trial) as the central target arrow. A fixation cross was displayed for 1,200 ms. After presenting a black screen for 500–1,000 ms, the arrow flanker task was presented for 1,500 ms or until a response was obtained. After presenting a black screen for 1,000 ms, the next flanker task began. Overall, participants completed 100 flanker trials. The flanker-interference effect (Eriksen and Eriksen, 1974) was defined as the difference in reaction times under the incompatible and compatible conditions (a greater difference indicates more interference).

#### Creative Task

The AUT was selected to measure divergent thinking. Participants were given 2 min per object to verbalize as many uses as they could. Two lists of objects were used for each experimental session (pre-test: bucket, shoe, newspaper; post-test: umbrella, can, paperclip) (Radel et al., 2015). The order of the lists was randomized. According to Guilford (1950), the test is scored in terms of fluency, flexibility, and originality. The fluency score was calculated as the number of responses; the flexibility score as the number of categories of responses; and originality as the frequency of occurrence of a given response among the participants. A response frequency percentage of less than 1% was given a score of 2; a frequency between 1 and 5% was given a score of 1; and a frequency of more than 5% was given a score of 0 (Radel et al., 2015). Two experienced creative field coders rated participants’ responses. They had satisfactory inter-rater reliability (Cronbach’s alphas: 1 for fluency, 1 for originality, 0.872 for flexibility).

For convergent thinking, we used the updated Chinese Compound RAT, compiled by Xu et al. (2015) (cf. Bowden and Jung-Beeman, 2003). Each problem in this test consists of three words chosen from the Modern Chinese Frequency Dictionary (1986). High-frequency words (mean frequency: 4,981.6 per million) were used to ensure that participants would understand them. Based on a preliminary test, 135 items were selected. From these, we selected 40 items for which the solution rates ranged from 40 to 65%. Twenty items were used for the pre-test and 20 for the post-test. This selection of Chinese Compound RAT problems had satisfactory internal consistency (Cronbach’s *α* = 0.897) and criterion validity. The solution rate was 66%, and the average response time was 3.79 s. The experiment was compiled using the E-prime program. Five items were used in a practice experiment. At the beginning of each trial, a 500-ms fixation is displayed at the center of the screen. Participants must press the space bar and immediately enter the RAT item in the next screen; then, they must think of an answer and say it aloud immediately after pressing the space bar. The screen automatically records the answer spoken by the participant and presents the correct answer. Finally, participants are asked to judge the answer displayed on the screen. If the correct answer is consistent with the answer spoken, they should press Q; if not, they should press P. If after 15 s, the participant is still not able to determine the answer, the program skips to the next question. A random interval of 100–500 ms is presented between two items.

#### Physiological Measures

The participants deposited salivary samples at −15, 0, 15 min (T2), 25 min (T3), and 40 min (T4) after the stress task. To control for stress induced by lab environment in subjects, the lower of the two samples between the first sample and the second sample was chosen as the baseline (T1) (McHugh et al., 2010). Saliva was collected using Salivettes® (Sarstedt 51.1534.500, Germany). All saliva samples were stored at −22°C and then thawed and centrifuged at 3000 rpm. The cortisol concentration and salivary alpha amylase were determined by enzyme immunoassay (Jianglai, China).

## Results

The physiological data, flanker task, and creativity scores were analyzed using univariate analysis of variance (ANOVA) with group (HTA, LTA) as the between-subjects factor and time (measurement time points) as within-subject factor. The ANOVA tested for the main effects of trait anxiety and presence/absence of stressor and their interaction. The Greenhouse-Geisser correction for non-sphericity was performed wherever appropriate. Bonferroni corrections were used to control for multiple comparisons. Partial-eta^2^ ($\eta_{p}^{2}$) is reported as a measure of effect size. Descriptive statistics and correlation analysis were conducted using SPSS.

### Physiological Results

**Figure 2:**
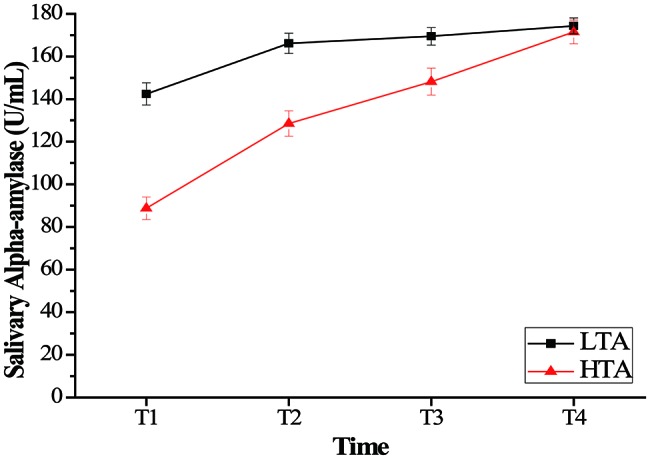
Mean salivary cortisol (nmol/L) as a function of time (minutes following intervention onset) for the stress. Error bars represent standard errors of the means.

For salivary cortisol, a repeated measurement ANOVA with the within-subject factor of time (T1, T2, T3, and T4) and between-subject factor of group (HTA and LTA) was computed with the salivary cortisol data to examine the effect of stress on salivary cortisol with high- and low-trait anxiety individual. ANOVA revealed a significant main effect of time, *F*(3,150) = 123.55, *p* < 0.001, $\eta_{p}^{2}$ = 0.712, and a significant main effect of group, *F*(1,50) = 7.75, *p* < 0.001, $\eta_{p}^{2}$ = 0.134. The HTA group (*M* = 7.45, SD = 2.23) was lower than the LTA (*M* = 8.36, SD = 1.77) group in salivary cortisol. The results also revealed a time × group interaction, *F*(3,48) = 6.88, *p* < 0.001, $\eta_{p}^{2}$ = 0.301. Bonferroni-corrected simple-effects tests at each time point revealed that the two groups showed significantly lower cortisol at T1 than at each of the subsequent times, (*p* < 0.001). Otherwise, compared with group difference in every time, the HTA group was significantly lower than the LTA group at T1 (*p* < 0.001) and T2 (*p* < 0.05) (see Figure 2).

**Figure 3:**
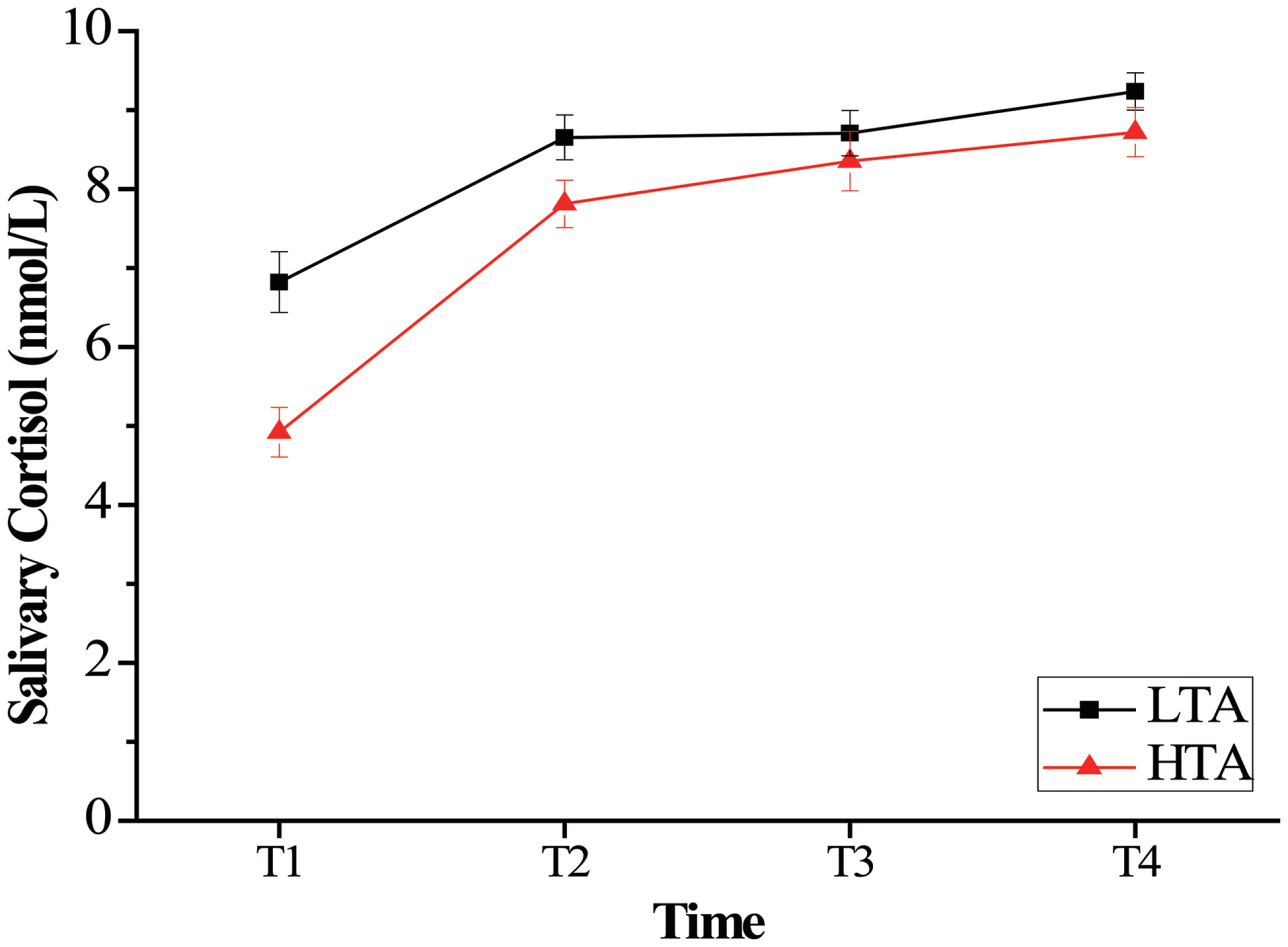
Mean salivary alpha amylase (U/ml) as a function of time (minutes following intervention onset) for the stress. Error bars represent standard errors of the means.

For salivary alpha amylase, a repeated measurement ANOVA with the within-subject factor of time (T1, T2, T3, and T4) and the between-subject factor of group (HTA and LTA) was computed for the salivary alpha amylase data to examine the effect of stress on salivary alpha amylase with high- and low-trait anxiety individuals. ANOVA revealed a significant main effect of time, *F*(3,150) = 114.16, *p* < 0.001, $\eta_{p}^{2}$ = 0.695, and a significant main effect of group, *F*(1,50) = 63.50, *p* < 0.001, $\eta_{p}^{2}$ = 0.599. The HTA group (*M* = 133.34, SD = 42.77) was lower than the LTA group (*M* = 164.27, SD = 26.61) in salivary alpha amylase. The results also revealed a time × group interaction, *F*(3,150) = 22.568, *p* < 0.001, $\eta_{p}^{2}$ = 0.311. Bonferroni-corrected simple-effects tests at each time point revealed that the two groups showed significantly lower cortisol at T1 than at each of the subsequent times (*p* < 0.001). Furthermore, compared with group difference in every time, the HTA group was significantly lower than the LTA group at T1, T2, and T3 (*p* < 0.001) (see Figure 3).

### Flanker Interference Effect Results

Descriptions of mean response times and error rates in the flanker task in the pre-test and post-test for the LTA and HTA groups are shown in Table 1. When analyzing the response times and error rate, extreme values of three standard deviations were excluded. Repeated measures ANOVAs with the within-subject factor measurement (pre-test and post-test) and the between-subject factor of group (HTA and LTA) were computed for the interference effect on response time and error rate. For interference effect in RTs, ANOVA yielded a significant interaction effect of group and measurement, *F*(1,50) = 20.38, *p* < 0.001, $\eta_{p}^{2}$ = 0.290. However, the main effect on group and measurement was insignificant (*p* > 0.05). Bonferroni-corrected simple-effects tests revealed that the HTA group (*M* = 81.13, SD = 40.21) was significantly slower than the LTA group (*M* = 54.19, SD = 32.25) in pre-test, *p* = 0.010. Nevertheless, the HTA group (*M* = 53.41, SD = 32.24) was significantly faster than the LTA group (*M* = 88.49, SD = 43.35) in post-test, *p* = 0.002. The HTA group pre-test (*M* = 81.13, SD = 40.21) was significantly slower than post-test (*M* = 53.41, SD = 32.24), *p* = 0.006, while the LTA group’s pre-test (*M* = 54.19, SD = 32.25) was significantly faster than the post-test (*M* = 88.49, SD = 43.35), *p* = 0.001 (see Table 1).

**Table 1:** Mean and standard deviations of response time (ms) and error rate (%) for flanker tasks pre and post stress for the LTA and HTA.

		Congruence		Incongruence		Interference effect	
		Pre-test	Post-test	Pre-test	Post-test	Pre-test	Post-test
RT	LTA	504.99 (80.86)	466.13 (87.54)	559.18 (78.87)	554.63 (101.61)	54.19 (32.25)	88.49 (43.35)
	HTA	574.16 (111.34)	558.23 (103.08)	655.29 (112.16)	611.65 (107.31)	81.13 (40.21)	53.41 (32.24)
ER	LTA	0.0054 (0.0190)	0.0054 (0.0190)	0.0788 (0.1384)	0.0300 (0.0763)	0.0735 (0.1429)	0.0246 (0.0782)
	HTA	0.0296 (0.0450)	0.0162 (0.0301)	0.1150 (0.2243)	0.0323 (0.0494)	0.0854 (0.2320)	0.0162 (0.0636)

**Figure 4:**
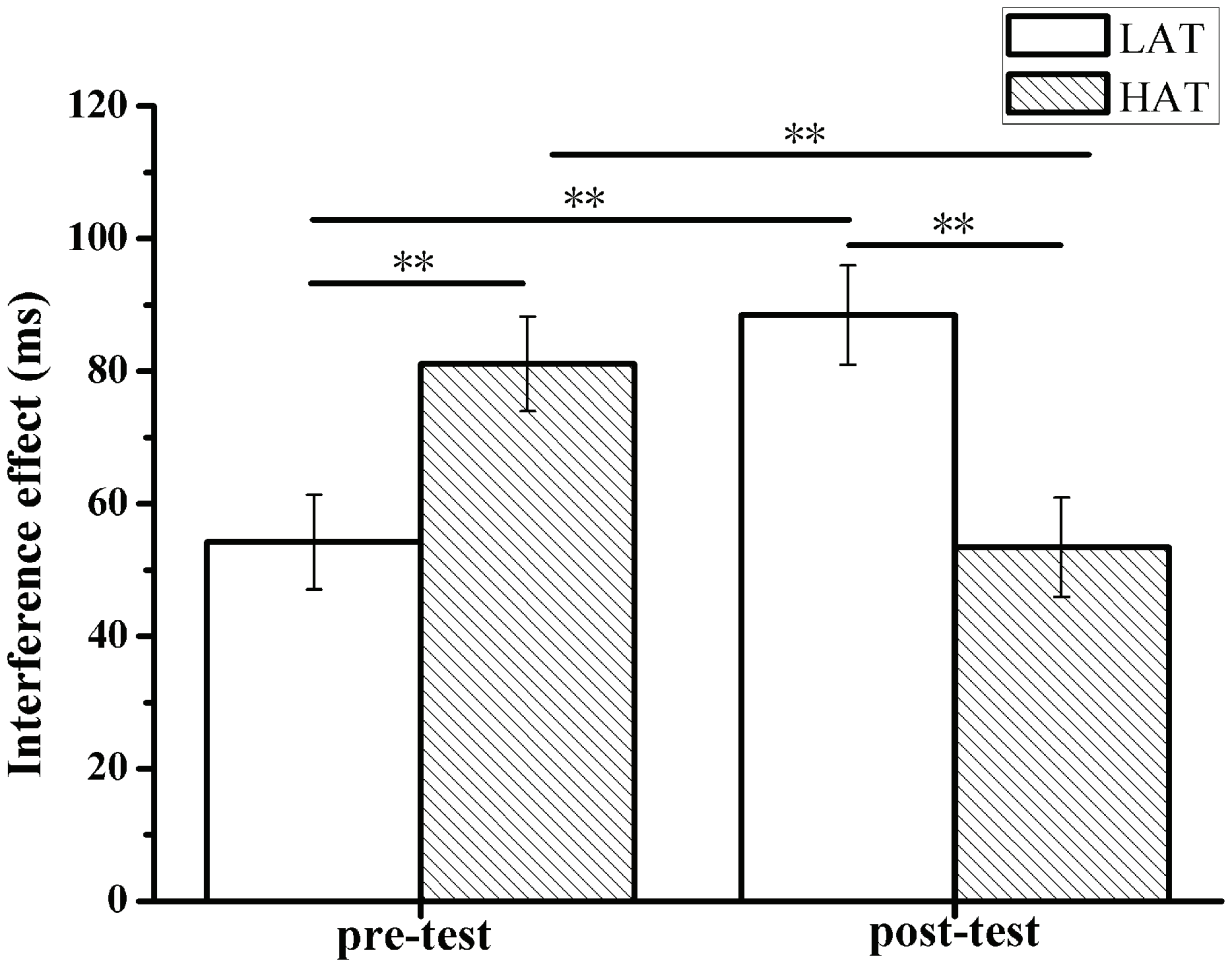
Interference effects (i.e., incongruent-congruent) on response time (a) pre- and post-TSST, for the HTA and LTA groups. Error bars represent standard errors of the means. ^**^ *p* < 0.01.

For the interference effect in error rate, ANOVA only yielded a significant main effect of measurement (pre-test and post-test); *F*(1,50) = 5.65, *p* = 0.021, $\eta_{p}^{2}$ = 0.102. Post-test (*M* = 0.020, SD = 0.013) showed significantly lower score than the pre-test (*M* = 0.079, SD = 0.036), while the main effect on group and the interaction effect between group and measurement were insignificant (*p* > 0.05) (see Figure 4).

### Creativity Results

Repeated measures ANOVAs for the within-subject factor measurement (pre-test and post-test) and between-subject factor of group (HTA and LTA) was computed for the AUT (fluency, flexibility, and originality) and RAT (response time and accuracy) to examine the effect of stress on creativity with high- and low-trait anxiety individuals.

**Figure 5:**
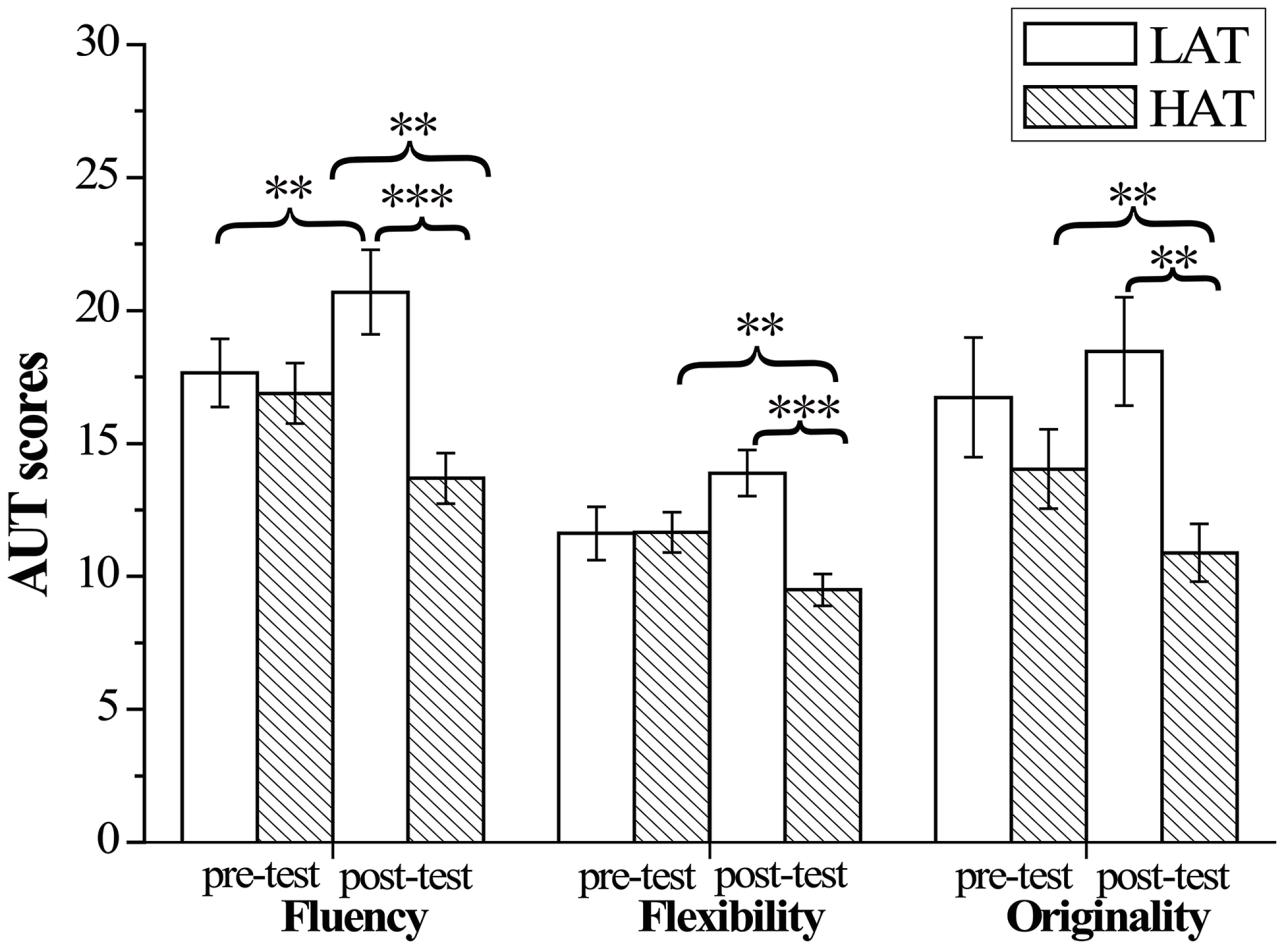
Mean and standard deviation for Alternative Uses Test scores for the HTA and LTA groups. Error bars represent standard errors of the means. ^**^ *p* < 0.01, ^***^ *p* < 0.001.

For *fluency* of AUT, the results only revealed a significant time × group interaction, *F*(1,50) = 16.29, *p* < 0.001, $\eta_{p}^{2}$ = 0.245. Bonferroni-corrected simple-effects tests revealed that the HTA group (*M* = 20.69, SD = 8.13) showed significantly more fluency than the LTA group (*M* = 13.69, SD = 4.86) only in post-test, *p* < 0.001. Interestingly, compared with group difference in pre- and post-test, the HTA group’s (*M* = 13.69, SD = 4.86) pre-test result was significantly lower than that of the post-test (*M* = 16.89, SD = 5.78), *p* = 0.005, whereas the LTA group scored significantly higher in pre-test (*M* = 20.692, SD = 8.13) than post-test (*M* = 17.65, SD = 6.56), *p* = 0.008. For AUT *flexibility*, the results revealed a significant time × group interaction, *F*(1,50) = 17.70, *p* < 0.001, $\eta_{p}^{2}$ = 0.261. Bonferroni-corrected simple-effects tests revealed that the HTA group (*M* = 9.50, SD = 3.05) showed significantly lower flexibility than the LTA group (*M* = 13.88, SD = 4.43) in the post-test, *p* < 0.001. Compared with group difference in pre- and post-test, HTA group’s pre-test score (*M* = 11.65, SD = 3.87) was significantly larger than that of their post-test (*M* = 9.50, SD = 3.05), *p* = 0.006, whereas the LTA group scored (*M* = 11.62, SD = 5.12) significantly lower in pre-test than post-test (*M* = 13.89, SD = 4.43), *p* = 0.004. For AUT *originality*, the results also revealed a significant time × group interaction, *F*(1,50) = 6.36, *p* = 0.015, $\eta_{p}^{2}$ = 0.113. Bonferroni-corrected simple-effects tests revealed that the HTA group (*M* = 10.88, SD = 5.55) showed significantly less originality than the LTA group (*M* = 18.46, SD = 10.39) in post-test, *p* = 0.002. Compared with group difference in pre- and post-test, HTA group’s score in pre-test (*M* = 14.04, SD = 7.61) was significantly higher than in post-test (*M* = 10.88, SD = 5.55), *p* = 0.025 (see Figure 5).

**Figure 6:**
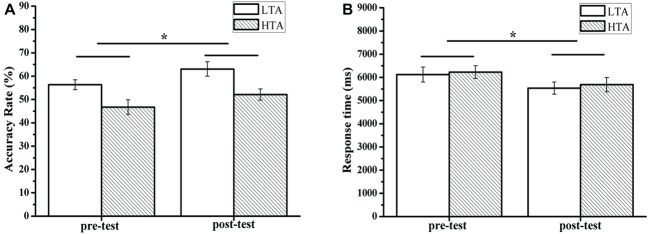
Mean accuracy **(A)** and response time **(B)** for the Remote Associates Test or for the HTA and LTA groups. Error bars represent standard errors of the means. ^*^ *p* < 0.05.

For the accuracy of the RAT, there was a significant main effect of time (pre-test and post-test), *F*(1,50) = 7.02, *p* = 0.011, $\eta_{p}^{2}$ = 0.123. The pre-test accuracy score (*M* = 51.54, SD = 14.30) was significantly lower than the post-test accuracy score (*M* = 57.60, SD = 15.03). The results showed a significant group effect, *F*(1,50) = 11.11, *p* = 0.002, $\eta_{p}^{2}$ = 0.182. The HTA group scored (*M* = 49.42, SD = 14.27) significantly higher than the LTA group (*M* = 59.71, SD = 13.84). There was no interaction effect between measurement and group. For the RAT response time, the results showed a main effect of time (pretest and posttest), *F*(1,50) = 5.00, *p* = 0.030, $\eta_{p}^{2}$ = 0.091. The pre-test score (*M* = 6174.26, SD = 1513.66) was significantly lower than the post-test score (*M* = 5610.49, SD = 1435.86) (see Figure 6).

To further verify whether there was a mediating mechanism in the process of stress affecting creativity using the bootstrapping method using PROCESS (Hayes, 2013) among flanker performances (model 4) (Preacher and Hayes, 2008). The 95% bias-corrected confidence interval (CI) was examined based on 1,000 bootstrap samples. The area under the curve with respect to increase (AUCi) was calculated using the trapezoidal method for HTA and LTA groups. Pruessner et al. (2003) pointed out that the method represented time-related changes and overall intensity of said changes in salivary cortisol and salivary alpha amylase levels. We performed *z*-transformed AUCi in sC and sAA data because of the individual differences in biological markers (sC and sAA), which were considered independent variables (sAAAUCi; sCAUCi). The changes in creative task performances were considered dependent variables (fluency, flexibility, and originality of the AUT; response time and accuracy of the RAT) and changes in flanker task performances were considered mediator variables (reaction time (FRT) and error rate interference effect (FEI) of flanker); meanwhile, the baseline of flanker and creative task performances were considered control variables. Table 2 presents the correlations among all variables.

**Table 2:** Correlation coefficients between biological, Flanker and creative performance.

Variables	1	2	3	4	5	6	7	8	9
1. sCAUCi	–	0.312^*^	−0.111	−0.282^*^	−0.178	0.016	0.108	0.031	−0.192
2. sAAAUCi		–	−0.521^**^	−0.480^**^	−0.433^**^	−0.153	−0.037	−0.238	−0.234
3. Fluency			–	0.755^**^	0.668^**^	−0.032	0.249	0.165	0.234
4. Flexibility				–	0.741^**^	0.103	0.115	0.299^**^	0.115
5. Originality					–	0.073	0.069	0.182	0.039
6. RT						–	−0.295^*^	0.225	−0.048
7. ACC							–	0.102	0.025
8. FRT								–	−0.078
9. FEI									–

* p < 0.05;

** *p < 0.01*.

**Figure 7:**
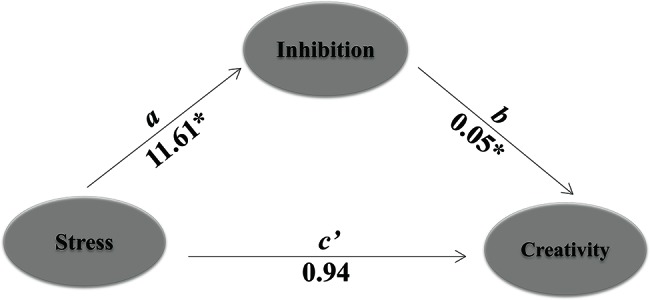
Mediation analyses of cognitive inhibition on the relationship between stress and creativity. Learning stress served as independent variable. Path *a* is the effect of stress on the proposed mediator, while path *b* represents the effect of the mediator on creativity. Path *c’* shows stress’s direct effect on creativity. Stress using the indictor of sAAAUCi; inhibition, FRT; creativity, fluency. Result presented here is based on 1,000 bootstrap samples. ^*^p < 0.05.

Results only showed that cognitive inhibition (reaction time interference effect of flanker) mediated the effect of stress (AUCi for sAA) on creativity (fluency of AUT), with an estimate of 0.59 and a 95% bootstrap CI of 0.0061–1.4137 (see Table 3, Figure 7). Based on this result, we claimed that cognitive inhibition was related to pre-post creativity performance (divergent thinking) in both HTA and LTA groups. Furthermore, this result supported the above results that the increase in inhibition control was associated with significantly decreased divergent thinking performance of the HTA group, while decrease in inhibition control was associated with significantly increased divergent thinking performance of the LTA group.

**Table 3:** Mediation results presented based on 1,000 bootstrap resamples.

	Direct effect *c´*	Path *a*	Path *b*	Indirect effect
FRT	−0.94	11.61^*^	0.05^*^	0.59 (0.0061–1.4137)^†^

*Adjusted coefficients with 95% confidence intervals*.

† *Indicates a 95% confidence interval that does not include 0. FRT represents the reaction time interference effect for Flanker task*.

* *p* < 0.05.

## Discussion

The present study examined the mechanism underlying the effect of acute stress on creative thinking, and to what extent this mechanism is influenced by individual differences in trait anxiety. We found higher levels of salivary cortisol and salivary alpha amylase after the TSST, indicating that participants experienced robust activation of the HPA and SNS. These results were in keeping with previous research, indicating that these two effects are likely due to different neural mechanisms, including the slowly increasing and persistent sC responses during HPA activation and the sAA responses during SNS system activation (Kirschbaum and Hellhammer, 1989; Foley and Kirschbaum, 2010). It is understandable that cognitive tasks (Flanker and creative task) also induced the increase in biological indicators in that cognitive stressors could produce subjective and objective stress effects (MacLeod, 1991; Renaud and Blondin, 1997). In present study, it is important to note that individuals were exposed to robust and reliable stress situation whatever the stress was induced by stress task or by the creative task itself.

One interesting finding was that the HTA group had lower salivary cortisol and salivary alpha amylase than the LTA group. This was consistent with earlier observations, which showed that HTA individuals (who have a relatively low baseline compared with the LTA group) often experience stress in daily social life. This was in line with another study showing that individuals with social anxiety disorder had a decreased cortisol baseline to stress (Elzinga et al., 2010). One possible explanation was that HTA individuals might initially present increased adrenocortical activity, leading to activation of chronicity compensatory mechanisms, gradually resulting in the attenuation in cortisol (Steudte et al., 2011). The repeated exposure to stress then results in habituation reactions, thereby reducing the individual HPA axis response level (Schommer et al., 2003; Shirotsuki et al., 2009). LTA individuals (who have a relatively high baseline compared with the LTA group), on the other hand, might be more effective in responding to environmental threats owing to their higher cortisol secretion (Villada et al., 2016).

As for the RT interference effect, compared with the LTA group, the HTA group showed worse inhibition control ability before stress, but significantly better inhibition control after. This was consistent with the ACT, which explains that HTA reduces inhibitory control compared with LTA (Eysenck et al., 2007). More importantly, under a stress manipulation, we saw a reduction in interference, whereas LTA individuals showed an increase due to induced anxiety. Combined with the theory of social promotion (Baron, 1986), individuals under stress could narrow the scope of attention and pay closer attention to the target information without distractors because these individuals had no remaining resources to deal with irrelevant information (Chajut and Algom, 2003).

For creative thinking performance, the mediation analysis showed that the increase in inhibition control was associated with significantly decreased divergent thinking performance in the HTA group, while a decrease in inhibition control was associated with significantly increased divergent thinking performance in the LTA group. These results partially supported the hypothesis and were consistent with previous observations, which indicated that anxiety traits modulate biological responses related to cognitive control and representation of cognitive improvement in individuals with HTA under stress (Sehlmeyer et al., 2010). However, the mediation analysis indicated no significant effects of inhibition control on convergent thinking.

According to the ACT, HTA might not decrease effectivity under certain conditions, thereby enabling HTA individuals to recruit additional processing resources to match those of LTA individuals (Eysenck and Derakshan, 2011; Berggren and Derakshan, 2013; Ward et al., 2017). In our study, HTA enhanced top-down processing and thereby hampered divergent thinking. For LTA, the decreased influence of bottom-up automatic processes was more helpful in associating remote ideas during the divergent thinking task (Chrysikou et al., 2014; Barr et al., 2015; Beaty et al., 2016; Hao et al., 2016; Kenett et al., 2018). The finding indicated that the trait anxiety moderates the effect of inhibition control on creativity under stress. The effect of inhibition on creativity also differed with the type of creativity. In other words, a low-inhibition state would enable individuals to obtain potentially useful information in a semantic network through a free association for divergent thinking; however, such a state would cause more distraction, hampering convergent thinking (Eysenck, 1995; Radel et al., 2015).

However, it was surprising that the HTA and LTA groups did not significantly differ in their RAT performance. Our study revealed that there was a shorter response time and a higher accuracy rate after stress in both trait anxiety groups. In the RAT, which is a measure of convergent thinking, stress induction was associated with higher accuracy and shorter reaction times. One possible explanation is that acute stress could increase individual dopamine levels (Robbins and Arnsten, 2009), which might help to facilitate creative problem-solving, such as the RAT (Cristofori et al., 2018). Besides, the problem solution for RAT could involve analytical strategies and insight strategies (Kounios and Beeman, 2014). In negative affect state, people are inclined to use analytical strategies and perform higher accuracy in high negative affect compared to relatively low negative emotions (Shen et al., 2019). Stress is usually accompanied by a relatively higher negative affect which prompts individuals to apply more analytical strategies to facilitate convergent thinking.

## Future Directions

Creative cognition involves recruitment of working memory (Chuderski and Jastrzębski, 2017, 2018), inhibition (Radel et al., 2015; Teng et al., 2018), and cognitive flexibility (Müller et al., 2016). Our findings indicated that acute stress impaired inhibitory control in LTA individuals but increased inhibitory control in HTA individuals. However, the mechanism underlying the effect of stress was no doubt very complex. Numerous studies showed that activation of the HPA axis was considered to have a significant impact on executive function (working memory, inhibition, and cognitive flexibility). Increased cortisol also could impair working memory (Shields et al., 2015), reduce cognitive inhibition, and increase response inhibition (Shields et al., 2016). In terms of cognitive flexibility, the general conclusion was that stress impaired cognitive flexibility (Alexander et al., 2007; Shields et al., 2016). Recent research has shown that HPA axis activation reduces switching flexibility but increases individual flexibility (Goldfarb et al., 2017). To better understand the role of cognitive control in creative thinking, we need a deeper understanding of the relationship between executive control components and acute stress.

We also found individual differences in creativity under stress. Future research should focus next on individuals with high and low creativity (Beaty et al., 2018), which would enable the assessment of brain functional connectivity as a predictor of individual creative ability under acute stress.

## Conclusion

The results showed that cognitive inhibition was influenced by trait anxiety and acute stress. Compared to before experiencing acute stress, there was a lack of cognitive inhibition in LTA individuals and they performed better on the AUT (fluency) after acute stress. HTA individuals, on the other hand, showed a decreased interference effect and reduced performance in the AUT (fluency, flexibility, and originality). In the RAT, there were shorter response times and increased accuracy after acute stress in both trait anxiety groups.

Thus, the findings suggest that cognitive control, which is modulated by changes in acute stress, influences creative cognition. The findings also indicated that acute stress can be influenced by anxiety, thus highlighting the crucial relation between creative cognition, acute stress, and individualdifferences.

## Author Contributions

HD and WH designed the experiments. XW and ZW carried out the experiments. FZ analyzed the experimental results. WX and YK assisted with performing the experiments. HD wrote the manuscript.

### Conflict of Interest Statement

The authors declare that the research was conducted in the absence of any commercial or financial relationships that could be construed as a potential conflict of interest.
